# Knowledge and attitudes relating to cervical and breast cancer among women in Maseru, Lesotho

**DOI:** 10.4102/phcfm.v14i1.3459

**Published:** 2022-12-14

**Authors:** Maseabata M. Ramathebane, Mopa A. Sooro, Richard M. Kabuya, Abdul-Rauf Sayed

**Affiliations:** 1 Department of Pharmacy, Faculty of Health Sciences, National University of Lesotho, Maseru, Lesotho; 2 Senkatana Oncology Clinic, Maseru, Lesotho; 3 Bristol Meyer Squibb Foundation, Cape Town, South Africa

**Keywords:** cross-sectional, breast cancer, cervical cancer, knowledge, attitudes, cancer screening, Lesotho

## Abstract

**Background:**

Cancer has remained one of the leading causes of death worldwide. In Lesotho, breast and cervical cancers contribute about 43% of all the cancer cases annually.

**Aim:**

This study is aimed at comparing knowledge, attitudes, and practices between breast and cervical cancers among females in Maseru.

**Settings:**

This study consists of women residing in five study sites which have clinics that offer cervical and breast cancer-screening services.

**Methods:**

A cross-sectional study was conducted in June 2021 in Maseru, the Capital city of Lesotho. The participants were interviewed using a pre-tested questionnaire, through which their knowledge, practices about, and attitudes towards breast and cervical cancers were assessed.

**Results:**

A total of 228 women aged 15–75 years participated in the study and the majority were aged 30 years and above. Of the women interviewed for cervical cancer, 89.5% had heard of it, 11.8% had heard of its screening, and 7.4% had at least one examination. Similarly, for breast cancer, 77.6% of women who had heard of it, 72.9% had heard of screening, and 40.1% of women did at least one examination.

**Conclusion:**

The majority of women were more knowledgeable about cervical cancer than breast cancer. However, more women had heard about breast cancer screening than cervical cancer screening. Therefore, there is a need for awareness campaigns related to cervical cancers’ screening.

**Contribution:**

There is an urgent need to intensify awareness about cervical and breast cancer screening and availability of services at the nearby clinics.

## Introduction

Cancer and other non-communicable diseases such as cardiovascular diseases have increasingly become some of the major causes of premature death globally.^1^ In 2020, new cancer cases were reported to be around 19.3 million globally and deaths reported were approximately 10 million.^2^ The most commonly diagnosed cancer is female breast cancer, where new cases were estimated at 2.3 million, thus making up all new cancer diagnoses to be approximately 11.7%.^3^ Breast cancer has been the leading cause of deaths in women globally, accounting for 684 996 deaths in 2020.^1^ Over 604 127, new cases of cervical cancer were reported in 2020 alone, and was the fourth most common cancer worldwide.^1,2^ In Lesotho, the most diagnosed cancer is cervical cancer with the incidence of 28.8% of all diagnosed cancers, followed by breast cancer with the incidence of 9.6% of all cancers diagnosed in 2020.^4^

In 2020, it was further noted that the most commonly diagnosed cancer worldwide is female breast cancer followed by lung cancer.^2,5^ An increase in the incidence and death from breast cancer have been reported, more especially in developing countries, the phenomenon which has been associated with increasing urbanisation.^6,7,8,9,10^ In addition, other related cancer-risk factors that play a significant role include: obesity, physical inactivity, and changes in reproductive patterns.^6,7,8,9,10^ Therefore, the lifetime risk of developing breast cancer differs by country and ethnicity because of the exposure to risk factors. Among women, breast cancers have evidence-based methods of prevention and early detection which can be employed even in low-resource countries.^2^ It has been established that the risk factors for breast cancers are mainly modifiable and include alcohol use, physical inactivity, and obesity.^8^ However, in relation to breast cancer, the three most important risk factors for women include: gender, age, and family history of breast or ovarian cancers.^10^ Other risks associated with breast cancer include genetic mutations of genes, such as BRCA1 and BRCA2.^11^ Exposure to drugs such as diethylstilbesterol and radiation therapy are also implicated in the promotion of breast cancer.^12^ Women with dense breasts have also been proven to have higher chances of developing breast cancer than average.^13^ As a way of ensuring and promoting breast health,^6^ individualised breast cancer risk assessment has been considered paramount.^14^

Research has shown a relatively low incidence of breast cancer among women in sub-Saharan Africa (SSA) compared to African–American women.^14^ This incidence has reportedly been attributed largely to a protective reproductive history of SSA women. The majority of women in SSA reach menarche late and menopause early. There is further high parity with prolonged breastfeeding. They experience irregular menses and fewer ovulatory cycles and these all contribute to the low incidence of breast cancer amongst these women.^13^ Despite such a low incidence among the SSA women, the majority of breast cancer cases were diagnosed at an advanced stage, resulting in higher mortality rates.^14,15^ Delay in patients’ seeking healthcare has been because of people’s ignorance of the early stages of and the stigma associated with the disease.^15^ Studies have further shown that women’s knowledge about and attitude towards the disease significantly influence their health-seeking behaviour.^16,17^

Practices such as breast self-examination and clinical breast examination have been shown to reduce breast cancer stage during diagnosis.^9,11^ However, more intensive screening such as performing a mammography, breast magnetic resonance imaging, breast ultrasound, or molecular breast imaging has been shown to be more effective especially for the detection of less aggressive cancers and cancers in women with dense breasts.^13,17^ Studies have shown that breast cancers mainly detected by the clinical breast examination are those presenting with aggressive features.^17^ Early diagnosis of breast cancers in symptomatic individuals is relatively simple to implement, with significantly less investment being required.^16,17^ Therefore, adequate knowledge is important to influence positive attitudes, beliefs, and practice regarding breast cancers.^16,17,18^

It was further noted that in developing countries, the cervical cancer-related deaths are almost 90%, and this is attributed to the fact that SSA, Central and South America, and South-Eastern Asia have the highest cervical cancer incidence and mortality rates.^19^ Chronic infections with human papillomavirus (HPV) have reportedly been the main risk factor for cervical cancer.^20,21^ This is made worse by sexual intercourse at early ages and, with multiple sexual partners, which increases the risk of contracting cervical HPV infection.^22,23^ In addition, higher parity, oral contraceptive use, HIV infection, and smoking are also associated with other high-risk HPV subtypes.^22^ It was observed that cervical cancer is mostly prevalent in regions with high HIV infection, such as SSA.^20,24^ Cervical cancer was the most common cancer diagnosed in 2020 in Lesotho.^4^ Key to the prevention of cervical cancer is the identification of the premalignant disease through cervical screen as well as vaccination against HPV.^24,25^ There is evidence of high acceptability of the HPV vaccine in SSA.^23^ With the availability of screening and the HPV vaccine as well as generally slow progression of the disease,^22^ cervical cancer is considered nearly completely preventable.

However, several studies have shown a gap in the knowledge, practices, and attitudes towards cervical cancer and breast cancer.^26^ While the majority of the participants have demonstrated knowledge about and positive attitudes towards these cancers, there have been noticeable poor health-seeking practices with a high incidence of advanced cervical and breast cancer on diagnosis.^26,27^ Knowledge about the risk factors of the diseases, the attitude towards these cancers as well as the availability and utilisation of the cervical, and breast cancer-screening services in Lesotho are crucial in the fight against these cancers. The Senkatana Oncology Clinic is a clinic established with the joint effort of the National University of Lesotho and the Senkatana Center of Excellence. The clinic is envisioned to provide cancer treatment as well as engaging in cancer awareness and screening campaigns among the citizens of Lesotho. The study was, therefore, aimed at assessing the baseline knowledge, attitudes, and practices of the residents around the clinic, with the purpose to encourage early diagnosis and treatment of these cancers.

## Methods

### Study design

The quantitative cross-sectional study design was adopted, and a structured questionnaire was used. The study was conducted in June 2021 in Maseru, the Capital city of Lesotho.

### Setting

The population in Maseru consist of mainly Basotho whose official languages are Sesotho and English. The majority of people live under poor conditions with food insecurity. Most of the people are job seekers who migrated from rural areas. They mostly live under rented housing with electricity, sanitation, and clean water.^28^ The literacy rate is 87.4% (95% women and 85% men), and Lesotho offers free primary education. About 38% of women and 59% of men are employed.^28^ The selected study sites have primary healthcare facilities that offer cervical and breast cancer screening. Village health workers offer cancer education to the inhabitants of the selected villages. Patients diagnosed with either cervical or breast cancer are referred to Senkatana Oncology Clinic for care and treatment.

### Study population and sampling strategy

The sample size was calculated for a larger study to determine the level of knowledge of selected cancers in both men and women (15–75 years) among household members in Maseru. We used the sample size formula for estimating a population proportion.^29^ The study population was thought to be approximately 40 000. The anticipated proportion of respondents with adequate knowledge of the selected cancers was be about 50% (*p* = 0.5). The minimum sample size required was 384 with a margin of error of 5% around the 95% confidence intervals. In order to gain sufficient statistical power, a sample of 500 participants was targeted.

Villages were selected from the areas served by four satellite clinics in the catchment area of the Senkatana Oncology Clinic in Maseru. One village was selected close to each clinic and a fifth village close to the oncology clinic itself. Convenience sampling was used to obtain approximately 100 participants from households in each village. Where there was more than one eligible person in the household, they were all interviewed. Household members that were not at home during data collection were excluded from the study. This study reports on the data obtained from the women in this sample.

### Data collection

The validated questionnaire to investigate knowledge and attitudes related to cervical and breast cancer in Lesotho was not available. The data collection tool consisted of 16 closed-ended (yes/no/not sure) questions that determined knowledge of risk factors of cervical cancer and breast cancer, respectively. Other validated questionnaires from Champion’s Health Belief Model Scale and Powe’s Fatalism Inventory (modified version) were adopted in order to formulate questions that determined attitudes.^26,29,30,31,32^ The questionnaire used a 5-point Likert scale (strongly disagree/disagree/not sure/agree/strongly agree) to measure attitudes related to cervical (19 questions) and breast cancer (16 questions).

In June 2021, the questionnaire was validated with seven colleagues from the Faculty of Health Sciences and Senkatana clinic. They checked the content of the questionnaire, construction of questions, and the time taken to complete. The data collection tool was translated into Sesotho. Trained interviewers administered the questionnaire to eligible participants in households within the five villages.

### Data analysis

Questionnaire data were captured in Epi Info 7 and exported to Stata (version 13.1) for statistical analysis. The code of ‘0’ was used for an incorrect or ‘not sure’ response and ‘1’ for a correct response when determining knowledge scores of cervical and breast cancer-related risk factors. A composite score was derived for each of the risk-factor questions. A respondent who was considered as knowledgeable was the one who attained a composite score greater than or equal to 50% (average and above or ≥ 50%).

Frequency tables were made out of categorical variables, while continuous variables were presented as descriptive measures, including mean and standard deviation or median and range. The association between the binary variables was tested using Odds ratios. Multiple logistic regression analysis was used for all analyses and the aim was to determine the association of possible predictors. A *p*-value of less than 0.05 and a 95% confidence interval that does not span unity was thus considered as a threshold of statistical significance.

### Ethical considerations

The National Health Research Committee of the Lesotho Ministry of Health of Lesotho, gave approval for the Knowledge, Attitudes and Practice (KAP) study and this number was allocated to the study ID 02-2021. Data collection commenced after approval of the study. Confidentiality was maintained throughout the study; anonymity was maintained from data collection to the end of the study. Informed consent forms were filled by participant prior to taking part in the study. Security of patients’ information was ensured by keeping data in a locked cabinet in the principal investigator (PI) office throughout the duration of the project and all study material will be kept for a period of five years after completion of the project.

## Results

### Demographic characteristics

A total of 228 women participated in this survey with the response rate of 100%. The median age of the participants was 31 (ranging from 15 to 75 years), with approximately half of them (55.7%) aged 30 years and older (Table 1). The majority of the women (51.3%) were single, separated, divorced, or widowed, and approximately one-third (32%) were employed or self-employed. Almost three-quarters (73.2%) of the participants had completed high school education. The majority of the participants were identified as Christians (97.4%).

**TABLE 1:** Demographic characteristics of the study participants (*n* = 228).

Variables	Number	%
**Age-group**		
< 20	30	13.2
20–29	71	31.1
30–39	50	21.9
40–49	26	11.4
50–59	22	9.7
60+	29	12.7
Total	228	100
**Educational level**		
None	6	2.6
Primary education	55	24.1
Secondary or High school	127	55.7
Technical	5	2.2
Diploma	18	7.9
University	17	7.5
Total	228	100
**Educational scale**		
≤ Primary education	61	26.8
≥ High school	167	73.3
Total	228	100
**Marital status**		
Married	110	48.3
Single	89	39.0
Divorced	4	1.8
Separated	8	3.5
Widowed	16	7.0
Cohabitating	1	0.4
Total	228	100
**Occupation**		
Employed	28	12.3
Self employed	45	19.7
Retired	6	2.6
Student	17	7.5
Unemployed	132	57.9
Total	228	100

Note: Median age 31; Median range = 15–17.

### Knowledge of cervical cancer

More than three-quarter of the women (89.5%) had heard of cervical cancer. Multiple logistic regression analysis was used to investigate the association between the outcome variable, ‘ever heard of cervical cancer (yes/no)?’ and selected demographic variables age, education, and occupation. Thirty-year old or older participants were more likely to have ever heard of cervical cancer (OR = 4.1; 95% CI 1.5–11.7; *p* = 0.007). However, educational level and occupational level were not significantly associated with awareness of cervical cancer.

Among the women familiar with cervical cancer (204/228), 11.8% had heard of cervical cancer screening and 7.4% had undergone a cervical cancer-screening examination. None of the women had ever heard of HPV. Approximately three-quarter of the participants gave the appropriate responses to four of the nine questions related to risk factors for cervical cancer as follows: cervical cancer is preventable (74%); having many different sexual partners is a risk factor (75%); an early onset of sexual activity is a risk factor (73.5%), and sexually transmitted infections (STIs) are a risk factor for cervical cancer (79.4%). Over half of the participants (52.9%) knew HIV as a risk factor for cervical cancer, while 58.8% knew smoking as a risk factor for cervical cancer (see Table 2).

**TABLE 2:** Knowledge of cervical cancer risk factors among participants who were aware of cervical cancer (*n* = 204).^26^

Questions to assess knowledge of risk factors for cervical cancer†	Number	%
Are you more likely to get cervical cancer if someone in your family has it?	76	37.3
Is HPV a risk factor for cervical cancer?	56	27.5
Is HIV a risk factor for cervical cancer?	108	52.9
Is smoking a risk factor for cervical cancer?	120	58.8
Is cervical cancer preventable?	151	74.0
Is having many different sexual partners a risk factor for cervical cancer?	153	75.0
Is giving birth to many babies a risk factor for cervical cancer?	84	41.2
Is early onset of sexual activity a risk factor for cervical cancer?	150	73.5
Is STIs a risk factor for cervical cancer?	162	79.4

*Source*: Questions were adopted from Chaka B, Sayed AR, Goeieman B, Rayne S. A survey of knowledge and attitudes relating to cervical and breast cancer among women in Ethiopia. BMC Public Health. 2018;18(1):1072. https://doi.org/10.1186/s12889-018-5958-8

HPV, human papilloma virus; STIs, sexually transmitted infections.

† , The correct response for these questions was ‘Yes’.

As described in the methodology earlier, when the composite score for knowledge was used, it was indicated that the median knowledge score for the risk factors of cervical cancer was 5 (range 0–9) out of a maximum possible score of 9.

### Knowledge of breast cancer

More than three-quarter (77.6%) of the women interviewed had ever heard of breast cancer. The multiple logistic regression analysis found a significant association between the outcome variable, ‘ever heard of breast cancer (yes/no)?’ and age. Thirty-year old or older participants were more likely to have ever heard of breast cancer (OR = 3.1; 95% CI: 1.5–6.3; *p* = 0.003). However, educational level and occupational level was not significantly associated with awareness of cervical cancer.

Among the women familiar with breast cancer (177/228), 72.9% had heard of breast cancer screening. Approximately two-third of the participants gave appropriate responses to only two of the seven questions on risk factors for breast cancer. However, the majority of women (65%) did not consider bigger breasts as a risk factor for breast cancer; on the other hand, 64.4% gave the correct response, and that physical exercises make it less likely of getting breast cancer. Less than half of the participants gave the appropriate response to five of the seven questions on risk factors for breast cancer (Table 3).

**TABLE 3:** Knowledge of breast cancer risk factors among participants who were aware of breast cancer (*n* = 177).^26^

Questions to assess knowledge of risk factors for breast cancer†	Number	%
Do you think inherited gene defects can cause breast cancer?	65	36.7
Do you think a diet with lots of animal meat is a risk factor for breast cancer?	60	33.9
Do you think alcohol is a risk factor for breast cancer?	64	36.2
Do you think bigger breasts are a risk factor for breast cancer?‡	115	65.0
Do you think you are more likely to get breast cancer if someone in your family has it?	77	43.5
Do you think exercise makes it less likely of getting breast cancer?	114	64.4
Do you think breast feeding makes it less likely of getting breast cancer?	72	40.7

*Source*: Questions were adopted from Chaka B, Sayed AR, Goeieman B, Rayne S. A survey of knowledge and attitudes relating to cervical and breast cancer among women in Ethiopia. BMC Public Health. 2018;18(1):1072. https://doi.org/10.1186/s12889-018-5958-8

† , The correct response for these questions was ‘Yes’;

‡ , The correct response for this question was ‘No’.

As described in the methodology, using the composite score for knowledge, the results showed that the median knowledge score for the risk factors of breast cancer was 3 (range 0–7) out of a maximum possible score of 7. Fewer than half (41.8%) of the women who were aware of breast cancer had above-average knowledge of risk factors for breast cancer.

### Assessing attitudes towards cervical cancer

Attitudes towards cervical cancer were assessed separately using 19 questions (Table 4). Three-quarter of the participants who had heard of cervical cancer agree or strongly agree to the thought of cervical cancer as ‘scary’ (75%) and 77.9% responded ‘cervical cancer would threaten a relationship with her spouse or sexual partner’. Majority of the women (76%) agree or strongly agree that any adult woman can develop cervical cancer and approximately half (53.4%) preferred a female health worker to conduct a cervical examination.

**TABLE 4:** Attitude assessment of cervical cancer among participants who had heard of cervical cancer (*n* = 204).^26^

Questions to describe attitudes related to cervical cancer	%				
	Strongly disagree	Disagree	Not sure	Agree	Strongly agree
My chances of getting cervical cancer in the next few years are high	16.2	15.7	30.4	26.0	11.8
I feel I will get cervical cancer some time during my life	18.6	17.7	33.3	22.6	7.8
The thought of cervical cancer scares me	8.8	9.3	6.9	34.3	40.7
Problems I would experience with cervical cancer would last a long time	6.4	11.3	31.4	37.3	13.7
Cervical cancer would threaten a relationship with my spouse or sexual partner.	3.9	5.9	12.3	36.8	41.2
If I developed cervical cancer, I would not live longer than 5 years.	12.3	21.6	36.8	19.1	10.3
Having cervical exams takes too much time	18.1	24.0	35.3	18.1	4.4
Having cervical exams is too painful	12.8	21.6	38.7	21.6	5.4
Healthcare workers doing cervical exams are rude to women	17.7	23.5	33.3	14.2	11.3
I have other problems more important than having cervical exams in my life	35.8	44.6	9.3	7.8	2.5
I am too old to have cervical exams regularly	43.1	39.2	8.3	7.8	1.5
There is no health centre close to my house to have cervical exams	51.0	35.8	8.3	4.4	0.5
If there is cancer development in my destiny, having cervical exams will not prevent it	10.3	14.7	21.1	41.7	12.3
I prefer a female health worker to conduct cervical exams	21.6	16.7	8.3	25.5	27.9
I will never have cervical exams if I have to pay for it	42.2	33.3	8.3	9.3	6.9
I would be ashamed to lie on a gynaecologic examination table and show my private parts to have a cervical exam	49.5	27.0	5.9	10.8	6.9
Any adult woman including you can develop cervical cancer	10.3	3.9	9.8	44.1	31.9
Cervical cancer is a disease for prostitutes	68.6	17.2	9.8	1.5	2.9
Talking to family/friends about symptoms of cervical cancers is embarrassing	45.6	34.3	6.4	10.8	2.9

*Source*: Questions were adopted from Chaka B, Sayed AR, Goeieman B, Rayne S. A survey of knowledge and attitudes relating to cervical and breast cancer among women in Ethiopia. BMC Public Health. 2018;18(1):1072. https://doi.org/10.1186/s12889-018-5958-8

### Assessing attitudes towards breast cancer

Attitudes towards breast cancer were assessed separately using 16 questions (Table 5). Approximately three-quarter of the women familiar with breast cancer have considered it as ‘scary’ (78.5%). Almost two-third responded that ‘the problems experienced with breast cancer would last a long time’ (70.1%) and ‘breast cancer would threaten a relationship with her spouse or sexual partner’ (62.7%).

**TABLE 5:** Attitude assessment of breast cancer among participants who had heard of breast cancer (*n* = 177).^26^

Questions to describe attitudes related to breast cancer	%				
	Strongly disagree	Disagree	Not sure	Agree	Strongly agree
My chances of getting breast cancer in the next few years are high	14.7	15.3	34.5	29.9	5.7
I feel I will get breast cancer some time during my life	19.2	16.4	33.9	26.6	4.0
The thought of breast cancer scares me	9.0	10.2	2.3	40.7	37.9
Problems I would experience with breast cancer would last a long time.	3.4	7.3	19.2	53.1	17.0
Breast cancer would threaten a relationship with my spouse or sexual partner.	12.4	14.1	10.7	33.9	28.8
If I developed breast cancer, I would not live longer than 5 years.	17.0	29.4	28.8	19.2	5.7
Having breast exams takes too much time	24.3	27.1	33.9	14.1	0.6
Having breast exams is too painful	31.6	24.9	29.9	11.3	2.3
Healthcare workers doing breast exams are rude to women	24.9	28.3	28.3	10.7	7.9
I have other problems more important than having breast exams in my life	43.5	47.5	2.3	4.0	2.8
I am too old to have breast exams regularly	53.7	33.9	2.3	10.2	0.0
There is no health centre close to my house to have breast exams	58.2	31.1	5.7	4.5	0.6
If there is cancer development in my destiny, having breast exams will not prevent it	9.0	14.7	19.2	46.3	10.7
I prefer a female health worker to conduct breast exams	25.4	20.3	2.8	37.9	13.6
I will never have breast exams if I have to pay for it	42.4	34.5	5.7	9.6	7.9
It is embarrassing to have my breasts examined	63.3	28.3	3.4	4.0	1.1

*Source*: Questions were adopted from Chaka B, Sayed AR, Goeieman B, Rayne S. A survey of knowledge and attitudes relating to cervical and breast cancer among women in Ethiopia. BMC Public Health. 2018;18(1):1072. https://doi.org/10.1186/s12889-018-5958-8

Less than half of the women (40.1%), who have heard of breast cancer, have had a breast cancer examination. Almost half of the women (49.2%) reported knowing how to do a breast-self-examination.

## Discussion

The data presented in this KAP study reveal an average age of the sampled population to be 30 years and above, who are Christian by religion, and are mostly single. The results of this study show that many women were aware of cervical and breast cancers. More than three-quarters of women (89.5% and 77.6%) reported to have heard of cervical cancer and breast cancer, respectively. Similar results were seen in the study conducted in Zimbabwe where 85% of the respondents had heard of these cancers.^27^ Thirty-year-old or older participants were more likely to have ever heard of these cancers and education level was not significantly associated with the awareness of these cancers. In the current study, awareness of cervical cancer was not significantly correlated to the level of education. Furthermore, the participants with secondary or higher educated were almost two times more likely to have heard of breast cancer than the rest of the participants. However, contradicting reports on level of education and knowledge on cancers have been noted. For example, some studies have reported a positive association between these two variables, while others observed a negative association. Interestingly, other studies have ruled out any association between the educational level and knowledge on cancers.^19,20,21^

The median knowledge score for the risk factors of cervical cancer compared to that of breast cancer was relatively higher – being five (5) out of the maximum score of nine (9) compared to three (3) out of the maximum score of seven (7). Comparing knowledge on the breast cancer risk factors and awareness about breast cancer, it was found out that more than half of the women had knowledge about risk factors and less than half were aware of breast cancer. These results are supported by the studies carried out in other African countries, where it was found that among women, there is relatively low overall knowledge about breast and cervical cancer risk factors.^26,27^ This is the most potent barrier to early diagnosis of these cancers. In addition, the studies undertaken in Nepal indicated that increasing awareness of risk factors results in improved cancer-screening behaviour and preventive cancer practice.^33^

In the present study, of all the women who were aware of cervical cancer, none knew about HPV, the feature which is the risk factor for cervical cancer. This finding warrants a need for further embarking on campaigns and developing materials geared towards informing about these cancers. HPV is key to the development of cervical cancer. Therefore, individuals who knew about cervical cancer should have been informed about it.^21,22^

Also noted in this study is the positive attitude of the respondents towards the diagnosis procedures of these cancers, the majority of whom reported a cervical/breast examination as taking short time. While the majority reportedly associated the examinations with no pains, others were reportedly uncertain. They (the latter) further disagreed with the statement ‘I have other more important problems than having a breast/cervical examination’. There is a relatively similar attitude of the respondents towards cervical cancer screening and breast cancer examination as, for both cancers more than 80% of the women disagreed with the statement, ‘I have other problems more important than having breast/cervical examination’. Similarly, according to the study conducted in Ethiopia, half of the respondents who had knowledge had the positive attitude towards cervical cancer screening.^34^ The majority of the respondents further reported that these cancers may threaten their relationships with their spouse or sexual partner. The respondents’ attitude was generally similar for both cancers.

Among the women familiar with breast cancer (*n* = 177), 72.9% reported to have ever heard of breast cancer screening and 40.1% have done breast cancer screening, while 49.2% reported to know how to do a breast self-examination. On the other hand, among those who were familiar with cervical cancer (*n* = 204), 11.8% reported to have ever heard of cervical cancer screening and 7.4% had undergone a cervical cancer-screening examination. These results prove that indeed, with greater awareness, there is associated practice. However, more than half of the respondent reported having never had a breast cancer examination. Respondents reported low practice for breast and cervical cancer. This corresponds to Lesotho demography and health survey 2016 that stated that in the past 12 months, screening of breast cancer was 10%, and Pap smear was 4% among women aged 15–49.^28^ Even lower practice was reported in the study conducted in Zimbabwe, where 96.2% of respondents had never undergone cervical cancer screening.^27^ Furthermore, the current study findings probably corroborate the studies which have reported a gap in the practice with the knowledge about and attitude towards the breast and cervical cancers.^8,15,26,27^ However, the current study proves that when the community is informed of the availability of screening services, they do use such services. Therefore, there is need for improvement of strategies to inform the community about breast and cervical cancer-screening services.

The limitation of the study was that data collection was carried out at the household level during the week when most workers were at work, in which case only the views of the unemployed counterparts and those who were off-duty were gathered for the study. It is worth noting that these participants would also be available for community education, but may not represent the entire study population.

The implication of the study is the fact that knowledge, attitudes, and practice regarding cervical and breast cancers were assessed showing clearly that knowledge about the cancers can be improved. This has positive results on screening which will lead to early diagnosis and treatment. In terms of health service, the findings imply that awareness about these diseases should be intensified, because cervical and breast screening are offered in all primary health facilities. As for policy, Lesotho needs to strengthen the newly formed national cancer registry to register all patients who are diagnosed with cervical or breast cancer to ensure early treatment and limit lost to follow up between screening, diagnosis, and treatment. Regarding future research, follow-up qualitative research should be carried out to reveal the real barriers that hinder cervical and breast cancer screening and determine strategies to overcome them.

## Conclusion

The study has found the majority of participants, particularly 30 years and older being relatively more knowledgeable about cervical cancer than breast cancer. The finding, therefore, necessitates more sensitisation campaigns so as to educate these communities on the two cancers. Despite being that knowledgeable about cervical cancer, the majority of the interviewed women reportedly lack knowledge of HPV as a risk factor for cervical cancer, thus further showing a need for awareness campaigns against the scourge. As such, noting currently high incidences of cervical cancer in Lesotho, the strategies of improving cervical cancer screening and HPV vaccination statistics among women are recommended by this study.
